# Influencing mechanisms of live streaming influencer characteristics on purchase intention: The mediating role of emotional trust

**DOI:** 10.1371/journal.pone.0322294

**Published:** 2025-04-29

**Authors:** Rong Zhou, Angathevar Baskaran

**Affiliations:** Faculty of Business and Economics, University of Malaya, Kuala Lumpur, Malaysia; UMass Amherst: University of Massachusetts Amherst, UNITED STATES OF AMERICA

## Abstract

Live streaming e-commerce emphasizes the role of live streaming influencers and the dynamic interactions between viewers and live streaming influencers. Utilizing data collected from 400 questionnaires, this study delves into the mechanisms through which characteristics of live streaming influencers influence consumer purchase intentions, with a focus on consumer emotional trust as a mediating variable as well as consumer education level, age, perceived risk, and live-stream engagement as moderating factors. The findings indicate that the traits of live streaming influencers have a positive effect on consumers’ intent to purchase. Emotional trust mediates the influence of live streaming influencer characteristics on purchase intention. Consumers’ educational level positively moderates the relationship between the professionalism and homogeneity of influencers and their purchase intentions, while it negatively moderates the relationship between influencers’ attraction and interactivity with purchase intentions. Additionally, the age of consumers positively moderates the link between the professionalism of influencers and purchase intentions, but negatively moderates the links between homogeneity, attraction, and interactivity of influencers and purchase intentions. Furthermore, both consumers’ educational level and age positively moderate the impact of emotional trust on purchase intentions. Lastly, perceived risk and live-stream engagement respectively exert negative and positive moderating effects on the influence of influencer professionalism and attraction on purchase intentions. The study contributes to influencer marketing theory by adopting an innovative approach to systematically investigate the collective influence of all four live streaming influencer characteristics (professionalism, homogeneity, attraction, and interactivity) within a comprehensive framework.

## 1. Introduction

Live streaming e-commerce is an e-commerce model centered on live streaming interactions [1]. Live streaming influencers within the live streaming e-commerce realm can be seen as a distinct category of social media influencers. They actively engage with audiences on platforms such as Tik Tok, providing their insights on various products and services, thereby influencing the purchase intentions of their viewers [2]. Live streaming e-commerce offers a distinctive transaction scenario characterized by high-frequency interactions, leading to a range of emotional trusts for consumers [1]. Notably, the influence mechanisms at play in live streaming e-commerce differ significantly from those in traditional offline commerce and conventional e-commerce. While the latter two predominantly emphasize physical sellers and e-commerce platforms, live streaming e-commerce shifts its focus toward the role of live streaming influencers and the dynamic interactions between viewers and live streaming influencers. Consequently, to thoroughly understand the influence mechanism of live streaming e-commerce, it is essential to examine the multifaceted effects of influencer attributes, consumer sentiment, and consumer traits on the propensity to purchase.

Within the dynamic realm of live streaming e-commerce, influencers expertly harness the power of visual and auditory channels to impart product knowledge, effectively evoking impulsive purchase intentions among their audience [3]. The presence of shared traits between the influencers and viewers acts as a catalyst, amplifying the drive to purchase [4]. The allure of live streaming influencers is paramount, as it significantly propels viewer purchase intentions [5]. Furthermore, live streaming influencers possess the unique ability to transmit emotions directly to viewers through interactive exchanges, a potent force in shaping purchase intentions. The emotional resonance established during these interactions can profoundly influence consumer decisions [1]. Despite the wealth of insights from existing research on how live streaming influencer characteristics sway viewer purchase intentions, there remains a notable gap. Studies often isolate single attributes of influencers, failing to adopt a systematic approach that synthesizes these attributes within an overarching framework.

The emotions of trust that consumers feel, especially those prompted by influencers, play a significant role in determining their willingness to make purchases [1]. Although previous research has pinpointed emotional trust as a crucial mediating factor in consumers’ buying choices [5–7], there is a noticeable gap in the literature regarding the integration of emotional trust into a holistic model that assesses how the attributes of live streaming influencers impact consumers’ intent to purchase. Moreover, consumers with different educational backgrounds and different ages perceive emotional trust differently [8], necessitating a deeper investigation into the subtle mechanisms by which emotional trusts influence purchasing intentions in this context.

In the context of live streaming commerce, consumer perceptions and interactions with influencers are shaped by their educational levels and age. Highly educated consumers are more swayed by professional influencers, trusting their product recommendations more and showing stronger purchase intent. If the influencer shares similarities with the consumer, this group is more likely to approve of the influencer and increase their buying intention [9]. The more educated a consumer is, the more rational they are, not making purchases blindly due to an influencer’s appeal but considering product quality and performance. Less educated consumers are more drawn to influencer interactions, gaining a sense of attention that boosts their trust and likelihood to buy [10]. Older consumers value product quality and practicality more. They tend to trust influencers who demonstrate high professionalism and rely on their expertise, enhancing their purchase intent. However, these consumers have clearer self - awareness and needs, focusing on actual product effects and quality, not similarities with influencers. Thus, influencer homogeneity has little impact on their purchase decisions [11]. Older consumers are more rational and objective, not buying products just because of an influencer’s appearance or temperament. Instead, they focus on product value and quality. Therefore, influencer attractiveness has minimal effect on their purchase intent. In the decision - making process, older consumers prefer detailed product information and professional reviews over interaction with influencers. They may rely more on other information sources like product reviews and user comments. Hence, influencer interactivity has little influence on their purchase decisions [12]. Current research has explored how consumers of different ages and education levels are influenced by influencers, leading to varying purchase intentions. However, there is a gap in the literature regarding the moderating role of age and education level on the relationship between live streaming influencer characteristics and purchase intention in the context of live e-commerce.

In the realm of influencer marketing, perceived risk, which entails anticipated negative outcomes such as commodity, influencer, platform, payment, and logistics risks, is typically elevated [13]. However, when consumers perceive lower risks, the positive influence of influencer professionalism, homogeneity with consumers in values and lifestyle, attractiveness, and interactivity on purchase intention is significantly enhanced [14]. Under conditions of low perceived risk, consumers are more inclined to trust and follow recommendations from professional, similar, attractive, and interactive influencers, leading to a heightened willingness to make purchases [15]. Engagement, characterized by emotional connection, time spent, follower number, and money spent, reflects consumers’ active involvement in live broadcasts. High engagement amplifies the positive effects of influencer professionalism, homogeneity, attractiveness, and interactivity on purchase intention [16]. Consumers with high participation levels value influencers’ expertise, are more receptive to recommendations from similar and attractive influencers, and prefer obtaining information through interaction, all of which boost their willingness to purchase [17]. Current research has explored how consumers with varying levels of perceived risk and engagement are influenced by influencers, leading to different purchase intentions. However, there is a gap in the literature regarding the moderating effects of perceived risk and live-stream engagement on the relationship between live streaming influencer characteristics and purchase intention in the context of live streaming e-commerce.

Drawing upon the foundation of social media influencer marketing theory, this paper constructs a model that investigates how live streaming influencer characteristics influence consumers’ purchase intentions. It aims to explore the mechanisms by which live streaming influencer characteristics affect consumers’ purchase intention, with consumer emotional trust as a mediating variable as well as consumer education level, age, perceived risk and live-stream engagement as the moderating variables, by utilizing primary data gathered through 400 questionnaires and analyzed through SPSS Process Marco.

This study makes notable theoretical contributions. Adopting an innovative approach, it systematically investigates the collective influence of all four live streaming influencer characteristics (professionalism, homogeneity, attraction, and interactivity) within a comprehensive framework, thus introducing a novel dimension. This is framework innovation. Additionally, this study is the first to introduce emotional trust as the mediating variable within the model of how live streaming influencer traits influence audience buying behavior. This is mediation variable innovation. Also, this study is the first to explore the potential moderating roles of variables like consumer education level, age, perceived risk and live-stream engagement within the model where live streaming influencer characteristics affect consumer purchase intentions. These are moderation variable innovations.

## 2. Literature review and theoretical foundation

### 2.1. Literature review

#### 2.1.1. Live streaming influencer characteristics.

Live streaming influencer characteristics are as follows: professionalism, homogeneity, attraction, and interactivity.

(a) Professionalism

The expertise of social media influencers plays a pivotal role in shaping their credibility, and this, in turn, significantly influences the behavior of their followers [18]. Expert influencers hold a distinct advantage over their counterparts who lack relevant knowledge, particularly in terms of their ability to motivate fans to purchase products [19]. Live streaming influencers actively engaged in live streaming e-commerce effectively transmit their professional knowledge about products through visual and auditory means, consequently eliciting impulsive purchase intentions in consumers [3]. Specifically, the live streaming influencer’s professional product introduction not only saves the audience’s time that would otherwise be spent comprehending the product independently but also fosters trust among the audience, thus significantly bolstering their purchase intentions [2].

(b) Homogeneity

The distinction between two dimensions of homogeneity is of paramount importance: identity homogeneity, which reflects shared social standing, and values homogeneity, indicative of aligned cognitive frameworks [20]. The perceived commonality between consumers and influencers significantly shapes the former’s identification on influencers [21]. The sense of identification fosters trust, which in turn often leads to the endorsement and uptake of the influencers’ recommended products [22]. In the context of live streaming e-commerce, the impact of influencer homogeneity is particularly pronounced, serving as a key driver in spurring viewers’ intent to purchase. When viewers perceive a congruence with the live streaming influencers in terms of identity and values, this perceived homogeneity not only enhances trust but also acts as a catalyst for purchase decisions, making it a critical factor in the realm of live commerce [4].

(c) Attraction

Attraction typically encompasses a range of characteristics, including a pleasing appearance, an attractive figure, a melodious voice, engaging conversational skills, and other appealing attributes [23,24]. The allure of influencers holds significant sway over consumers’ purchase intentions, particularly in cases where consumers possess a desire to imitate [25]. Specifically, the physical attractiveness of influencers can amplify the trust of their followers, thereby exerting an impact on followers’ purchasing behavior [26]. In live streaming e-commerce, live streaming influencer attraction plays the primary role in driving viewers’ purchase intentions [5].

(d) Interaction

Influencer interaction denotes the mutual and dynamic exchange of information and engagement between an influencer and their audience. Within the context of live streaming e-commerce, this concept is embodied by the establishment of immediate and interactive communication pathways between live stream influencers and their viewership. The interactive nature of live streaming e-commerce serves as a powerful tool for fostering a deeper connection between influencers and consumers. By actively participating in the live discussions, influencers demonstrate a level of authenticity and approachability that can enhance the viewers’ sense of trust. As this bond strengthens, so does the likelihood of viewers transforming their interest into actual purchases [27].

Consequently, the paper puts forth a series of hypotheses (Hypotheses 1a, 1b, 1c, and 1d):

*Hypothesis 1a: Live streaming influencer professionalism could positively influence consumer’s purchase intention.*
*Hypothesis 1b: Live streaming influencer homogeneity could positively influence consumer’s purchase intention.*
*Hypothesis 1c: Live streaming influencer attraction could positively influence consumer’s purchase intention.*
*Hypothesis 1d: Live streaming influencer interactivity could positively influence consumer’s purchase intention.*

#### 2.1.2. Emotional trust.

The characteristics of live streaming influencers can elicit emotional trust [1], which have a profound impact on their propensity to make purchases. Consumers’ emotional trust is founded on their perceived trust in online sellers [28], and this emotional trust stimulates their willingness to purchase.

Professional influencers can demonstrate profound knowledge, rich experience, and professional skills [3]. When introducing products, they can accurately convey the features, advantages, and usage methods of the products, making consumers feel their professionalism and reliability [19]. Once consumers trust the professionalism of influencers, they will be more inclined to believe that the recommended products are of high quality [18]. This trust can reduce consumers’ doubts and risk perception in the purchasing decision-making process, thereby enhancing their willingness to buy [5].

Homogeneity refers to the similarities between influencers and consumers in certain aspects, such as age, gender, hobbies, and lifestyle [20]. When consumers find similarities with influencers, they will feel a sense of closeness and identification [21]. This similarity makes consumers think that influencers understand their needs and preferences better, thus making it easier to establish a trust relationship [22]. The trust generated based on homogeneity will make consumers more willing to accept the recommendations of influencers [4]. They believe that the products recommended by influencers are more in line with their taste and needs, so the willingness to buy will increase accordingly [29].

Attraction includes the influencer’s appearance, temperament, and personal charm [23,24]. Influencers with attraction can attract consumers’ attention and give them good impression [30]. This impression will make consumers more willing to approach influencers emotionally, thereby generating a certain degree of trust [25]. The trust brought by attraction is relatively superficial, but it can also affect consumers’ willingness to buy to a certain extent [5]. Consumers may be willing to try the products they recommend because of this emotional trust, especially when the products match the attraction of the influencer [26].

Interactivity refers to the degree of communication and interaction between influencers and consumers. In the live broadcast process, influencers can respond to consumers’ questions, comments, and feedback in time, making consumers feel cared for and valued. This interaction can enhance the emotional connection between consumers and influencers, making consumers trust influencers [27]. The trust brought by interactivity will make consumers more actively participate in the live broadcast activities and have a higher interest in the products recommended by influencers. In the interaction with influencers, consumers can understand the product information more comprehensively, thereby reducing the uncertainty of purchase, and then enhancing the willingness to buy [1].

In summary, Hypotheses 2a, 2b, 2c and 2d are proposed

*Hypothesis 2a: Emotional trust plays a mediating role in the impact of live streaming influencer professionalism on purchase intentions.*
*Hypothesis 2b: Emotional trust plays a mediating role in the impact of live streaming influencer homogeneity on purchase intentions.*
*Hypothesis 2c: Emotional trust plays a mediating role in the impact of live streaming influencer attraction on purchase intentions.*
*Hypothesis 2d: Emotional trust plays a mediating role in the impact of live streaming influencer interactivity on purchase intentions.*

#### 2.1.3. Education level and age.

In the realm of live streaming commerce, consumers with varying levels of education exhibit distinct perspectives towards live streaming influencers. Consumers with a higher level of education tend to have stronger information - screening and judgment abilities. They pay more attention to the professional knowledge and authority of influencers. When an influencer demonstrates a high level of professionalism, highly - educated consumers are more likely to be impressed by their professional knowledge, thus developing a higher degree of trust in the products recommended by the influencer, and further enhancing their purchase intention. In addition, consumers with a higher level of education usually have a clearer self - awareness and demand positioning. They are more likely to identify with influencers who share similar values and lifestyles with them. When there is homogeneity between the influencer and the consumers in these aspects, highly - educated consumers will think that the influencer better understands their needs, and thus are more willing to accept their recommendations, and the purchase intention will increase accordingly [31]. Moreover, consumers with a lower level of education are more likely to purchase products recommended by influencers because they like the influencers’ appearance or temperament. However, consumers with a higher level of education are more rational and objective. They will not blindly buy products just because of the influencer’s attractiveness, but will comprehensively consider factors such as product quality and performance. Finally, consumers with a lower level of education may be more easily attracted by the interaction of influencers. Through communication and interaction with influencers, they can get a sense of being noticed and valued, thus enhancing their trust in the influencer and purchase intention. On the contrary, for highly - educated consumers, if the interaction with the influencer fails to provide valuable information or solve their problems, they may question the professionalism and credibility of the influencer, and thus reduce their purchase intention [32].

Younger and older consumers hold distinct perspectives when it comes to live streaming influencers. Older consumers usually attach more importance to product quality and practicality. Therefore, they are more inclined to trust influencers who demonstrate a high level of professionalism. During the purchasing decision - making process, these consumers rely more on the professional knowledge and experience of influencers, thereby enhancing their purchase intention [33]. However, older consumers usually have a clearer self - awareness and demand positioning. They focus more on the actual effects and quality of products rather than the similarity in lifestyle or values between the influencer and themselves. Therefore, the homogeneity of influencers has little impact on the purchase intention of these consumers. Older consumers are generally more rational and objective. They do not buy products just because of the influencer’s appearance or temperament. Instead, they pay more attention to the actual value and quality of products [11]. Therefore, the attractiveness of influencers has little impact on the purchase intention of these consumers. During the purchasing decision - making process, older consumers pay more attention to detailed product information and professional evaluations rather than the interaction with influencers. They may be more inclined to obtain information through other channels (such as product reviews, user comments, etc.) rather than relying on the interaction with influencers [34]. Therefore, the interactivity of influencers has little impact on the purchase intention of these consumers.

Emotional trust affects purchase intention [1], but the impact is different for consumers with different educational backgrounds. Consumers with a high level of education often have stronger information - collection and analysis capabilities. They can obtain relevant information about products or services from multiple channels and critically think about this information. In this process, emotional trust plays a key role in screening information. If they have already established emotional trust in a certain brand, when faced with a large amount of complex information, this emotional trust will make them more likely to pay attention to and recognize information that is beneficial to the brand, thus strengthening their purchase intention. The values and cognitive patterns of highly - educated consumers are relatively more mature and stable. In their purchasing decisions, they consider not only the functional value of the product but also the culture and social values represented by the brand. If they have established emotional trust in a brand because it aligns with their values, this emotional trust will greatly enhance their willingness to purchase this brand because, in their view, the purchasing behavior is a support for the values advocated by the brand [12].

Meanwhile, the impact of emotional trust on purchase intention is different for older consumers and younger consumers. Older consumers usually have more extensive consumption experience. During years of consumption, they may have developed long - term emotional trust in certain brands. This emotional trust has a significant impact on their purchase intention. As they grow older, they rely more on this familiar emotional trust to make purchasing decisions because past consumption experiences have made them believe that the brands they trust can meet their needs [8]. Older consumers are more sensitive to risk perception. When purchasing products or services, they are more likely to choose brands that can bring them a sense of security. Emotional trust becomes an important factor in reducing risk perception in this process. Moreover, as they age, their consumption preferences gradually stabilize. Once they develop emotional trust in a certain brand, this preference will be continuously reflected in subsequent purchasing behaviors [35].

Consequently, hypotheses 3a and 3b are proposed

*Hypothesis 3a: Consumer education level moderates the relationship between live streaming influencer characteristics and consumers’ purchase intention.*
*Hypothesis 3b: Consumer age moderates the relationship between live streaming influencer characteristics and consumers’ purchase intention.*
*Hypothesis 3c: Consumer education level moderates the relationship between emotional trust and purchase intention.*
*Hypothesis 3d: Consumer age moderates the relationship between emotional trust and purchase intention.*

#### 2.1.4. Perceived risk and live-stream engagement.

Perceived risk encompasses the anticipated negative consequences associated with acquiring a specific product or service [36]. In the context of live streaming shopping, consumers often perceive a heightened level of risk. This perception is rooted in five primary categories of risk, namely commodity risk, live streaming influencer risk, platform risk, payment risk and logistics risks [37]. When consumers perceive lower risks, the positive impact of influencers’ professionalism on purchase intention becomes more significant. This is because consumers are more willing to trust recommendations from professional influencers under low-risk perception, thereby enhancing their willingness to purchase. When the perceived risk is low, the homogeneity between influencers and consumers in terms of values and lifestyle has a more significant positive impact on purchase intention. Consumers are more likely to accept recommendations from influencers who are similar to themselves under low-risk perception [13]. When the perceived risk is low, the attractiveness of influencers has a more significant positive impact on purchase intention. Consumers are more likely to purchase products recommended by influencers they find attractive in terms of appearance or temperament under low-risk perception. When the perceived risk is low, the interactivity of influencers has a more significant positive impact on purchase intention. Consumers are more willing to obtain information through interaction under low-risk perception, thereby enhancing their willingness to purchase [15].

Live-stream engagement refers to the degree to which consumers actively engage in interaction and communication during the live broadcast process and can be parsed into four distinct dimensions, namely emotional connection, viewing time, follower number and money spent [16]. When consumers have a high level of participation in live broadcasts, the positive impact of influencers’ professionalism on purchase intention becomes more significant. Consumers with high participation levels place greater emphasis on the professional knowledge and experience of influencers, thereby enhancing their willingness to purchase. When the participation level in live broadcasts is high, the homogeneity between influencers and consumers in terms of values and lifestyle has a more significant positive impact on purchase intention. Consumers with high participation levels are more willing to accept recommendations from influencers who are similar to themselves [38]. When the participation level in live broadcasts is high, the attractiveness of influencers has a more significant positive impact on purchase intention. Consumers with high participation levels are more willing to purchase products recommended by influencers they find attractive in terms of appearance or temperament. When the participation level in live broadcasts is high, the interactivity of influencers has a more significant positive impact on purchase intention. Consumers with high participation levels are more willing to obtain information through interaction, thereby enhancing their willingness to purchase [17].

Consequently, hypotheses 4a and 4b are proposed

*Hypothesis 4a: Consumer perceived risk moderates the relationship between live streaming influencer characteristics and consumers’ purchase intention.*
*Hypothesis 4b: Consumer live-stream engagement moderates the relationship between live streaming influencer characteristics and consumers’ purchase intention.*

### 2.2. Theoretical foundation

#### 2.2.1. Stimulus-Organism-Response model.

The Stimulus-Organism-Response (S-O-R) model is an improvement on the traditional stimulus-response model, taking into account the mediating role of individual psychological states between environmental stimuli and behavioral responses [39]. Stimuli are any changes or events in the external environment detected by an individual’s sensory organs. The organism represents the individual’s internal processes, such as emotional trust, which occur between the reception of stimuli and the final response. The response is the observable behavior after the internal processing of the stimulus. This response can be emotional (e.g., feeling satisfied or frustrated) or behavioral (e.g., purchasing a product or leaving a store). The S-O-R model extends the traditional stimulus-response model by placing the internal processes of the organism as a mediator between environmental stimuli and behavioral responses.

In the context of e-commerce live streaming, the application of the Stimulus-Organism-Response (S-O-R) model can help analyze the complex relationship between live streamer characteristics, consumer emotional trust, and purchasing behavior. The stimuli in e-commerce live streaming are mainly the characteristics of live streamers, including professionalism, homogeneity, attractiveness, and interactivity. The organism component refers to the consumer’s internal emotional state. When exposed to the characteristics of live streamers, consumers may experience emotional trust. These internal states are shaped by the consumer’s personal perception, past experiences, and the immediate context of the live streaming session. The response is the consumer’s willingness to purchase, which is the result of the interaction between the stimulus factors and the organism [1].

#### 2.2.2. Selectivity hypothesis.

The Selective Hypothesis posits that consumers with different levels of education adopt different strategies in processing information [40]. Specifically, consumers with higher levels of education typically have stronger information processing and analytical abilities, enabling them to more effectively filter and evaluate information, thereby making more rational decisions. In contrast, consumers with lower levels of education may have relatively weaker information processing abilities and may be more easily influenced by advertisements and promotional messages [31].

The Selective Hypothesis also suggests that consumers of different ages adopt different strategies in processing information [33]. Specifically, there are significant differences in information processing abilities and preferences among consumers of different age groups, which affect their purchasing decisions and behaviors. Younger consumers typically prefer to use digital channels and social media to obtain information. They are more adept at handling large amounts of information and are more willing to engage and participate to obtain product information. Older consumers typically place more emphasis on the practicality and cost-effectiveness of products. They are more likely to make purchasing decisions based on detailed product information and user reviews [34].

#### 2.2.3. Perceived risk theory and engagement theory.

Perceived risk theory is an important theory in consumer behavior. This theory suggests that consumers perceive various risks during the purchasing decision-making process, including financial risk, performance risk, time risk, physical risk, social risk, and psychological risk [36]. Perceived risk refers to the consumer’s subjective assessment of the potential negative consequences of a purchasing action. The level of perceived risk affects consumer purchasing decisions; high perceived risk can reduce the willingness to purchase, while low perceived risk can enhance it [37].

Engagement theory has wide applications in consumer behavior. This theory posits that the more time consumers invest and the more effort and energy they put in, the more likely they are to make a purchase [16]. Engagement can be divided into behavioral engagement, emotional engagement, and cognitive engagement. Behavioral engagement refers to the specific actions consumers take during the purchasing process, such as browsing, commenting, and purchasing. Emotional engagement refers to the emotional investment consumers have in a brand or product, such as brand loyalty and emotional connection. Cognitive engagement refers to the information processing and decision-making processes consumers undergo during the purchasing process, such as information search and evaluation [38].

**Fig 1:**
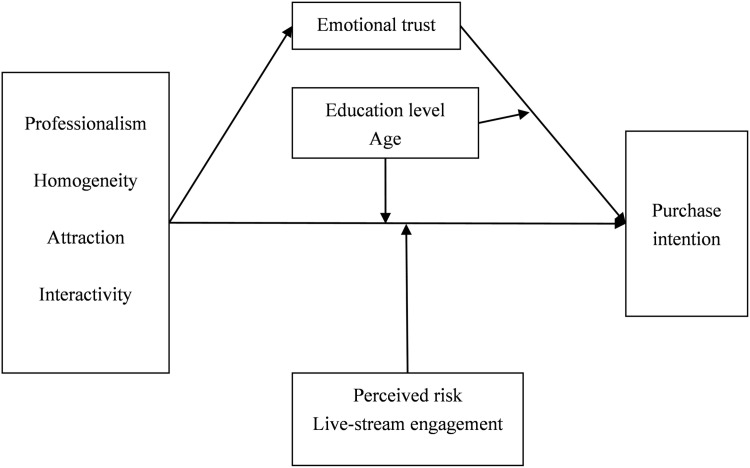
Conceptual model.

Based on the theoretical view, Fig 1 shows the relationships between the constructs and the respective hypotheses.

## 3. Research design

### 3.1. Questionnaire design

The initial segment of this study is dedicated to a specific target demographic: consumers who have made purchases through live streaming e-commerce platforms. This approach contrasts with that of Meng, Duan [1], who employed a virtual scenario. The rationale behind this choice is the anticipation that consumers with prior live streaming e-commerce purchasing experience may offer unique insights distinct from those who have not engaged in this mode of commerce. Consequently, the first inquiry is as follows: ‘*Have you ever made a purchase through live streaming e-commerce?*’ Should a respondent answer ‘*NO’*, the survey will be concluded for that participant. It’s noteworthy that this study focuses exclusively on Chinese consumers, a deliberate decision intended to mitigate the influence of cultural variations.

The survey questions for gender and age are modeled after the framework proposed by Law and Ng [12]. The question related to education level encompasses all possible education levels. Income is an essential factor to consider. For the monthly income item, a set of twelve options is provided to account for the diversity within the respondent groups: *Under 500*, *500–1000*, *1000–1500, 1500–2000*, *2000–2500*, *2500–4000*, *4000–6000, 6000–8000, 8000–10000, 10000–12000, 12000–14000* and *Above 14000* [41]. Shopping frequency, as it pertains to shopping habits, is modeled after the framework provided by Blake et al. (2003).

The final section of the questionnaire represents the core segment, requiring participants to consider their most recent purchase experience through live-streaming e-commerce. For this portion, a five-level Likert scale is employed to assess respondents, where ‘1’ corresponds to ‘strongly disagree’, ‘2’ signifies ‘disagree’, ‘3’ reflects ‘neutrality’, ‘4’ denotes ‘agree’, and ‘5’ indicates ‘strongly agree’. These rating scales have been adopted from established and well-regarded scales used by other researchers in the field, ensuring the reliability and consistency of the data collected.

As all the participants in this study are Chinese, it is imperative to have the questionnaire accurately translated into Chinese. To ensure linguistic precision and clarity, a team of experts proficient in both Chinese and English has meticulously reviewed the translated questionnaire. As a token of gratitude for their time and contributions, each participant received a modest incentive of 5 RMB upon the completion of the questionnaire. The questionnaire and methodology for this paper was approved by the Human Research Ethics Committee of the University of Malaya (Ethics approval number: UM.TNC2/UMREC_2335).

Sampling process is as follows. The sampling process for this study begins with defining the sample population, which includes 469 million Chinese consumers who have made purchases on live-streaming e-commerce platforms [42]. The sample size is meticulously calculated to exceed 385 participants, a figure that guarantees a 95% confidence level with a 5% margin of error, thus meeting the rigorous standards of social science research reliability. A reputable market research firm was appointed to manage the questionnaire administration, with the data collection phase taking place between 4 March 2023 and 4 June 2023. The sampling method is convenience sampling. The research team distributed 420 questionnaires across live-streaming e-commerce platforms and is able to collect the same number, achieving a perfect 100% response rate. Participants provide written informed consent. If participants are minors, consent from parents or guardians are provided. After rigorous review, 400 responses are validated, leading to a high effective return rate of 95.24%; responses are deemed invalid if participants had not actually made purchases on the platforms or if the provided information was incomplete.

### 3.2. Variable measurement

Based on the above literature review, the scales of variables are summarized in Table 1.

**Table 1:** Summary of variables scales.

Variables	Number	Measurement Index	Reference
Professionalism (A)	A1	When you watch the live broadcast, you think the live streaming influencer has professional knowledge	Xu, Cui [2]
	A2	When you watch the live broadcast, you think the live streaming influencer knows a lot about the recommended product	
	A3	When you watch the live broadcast, you think that the live streaming influencer has rich experience in using the recommended products	
Homogeneity (B)	B1	When you watch the live broadcast, you think the live streaming influencer is similar to your personality and taste	Zhou and Tong [4]
	B2	When you watch the live broadcast, you think the live streaming influencer shares your values	
	B3	When you watch the live broadcast, you think that the live streaming influencer’s interest in products is similar to yours	
Attraction (C)	C1	The reason you watch live broadcast is because you are attracted by the appearance of the live streaming influencer	Zhong, Zhang [5]
	C2	The reason you watch live broadcast is because he or she is charming	
	C3	When you watch the live broadcast, you think the live streaming influencer is humorous	
Interactivity (D)	D1	When you watch the live broadcast, you think the live streaming influencer has good interaction with you	Meng, Duan [1]
	D2	When you watch the live broadcast, you think the live streaming influencer can engage you deeply	
	D3	When you watch the live broadcast, you think the live streaming influencer can communicate with the audience in time	
Emotional trust (E)	E1	You think the live content of live streaming influencers is credible	Trivedi and Sama [19]
	E2	You think that live streaming influencers are worthy being trusted	
	E3	You think the products recommended by the live streaming influencers are reliable	
Perceived risk (F)	F1	When you watch the live broadcast, you feel the commodity risk	Li, Wang [37]
	F2	When you watch the live broadcast, you feel the live streaming influencer risk.	
	F3	When you watch the live broadcast, you feel the platform risk.	
	F4	When you watch the live broadcast, you feel the payment risk.	
	F5	When you watch the live broadcast, you feel the logistics risk.	
Live-stream engagement (G)	G1	When you watch the live broadcast, you perceived attachment to this live streaming influencer.	Hilvert-Bruce, Neill [16]
	G2	When you watch the live broadcast, you spend much time.	
	G3	When you watch the live broadcast, you follow the live streaming influencer.	
	G4	When you watch the live broadcast, you spend much money buying goods.	
Purchase Intention (H)	H1	You are very likely to consider buying products recommended by this live streaming influencer	Zhou and Tong [4]
	H2	You are willing to buy products recommended by this live streaming influencer	
	H3	You would recommend products recommended by this live streaming influencer to others	

### 3.3. Descriptive analysis

A descriptive statistical analysis is performed in Table 2 based on 400 valid questionnaires.

**Table 2:** Demographic characteristics.

Variables	Item	Frequency	Percent
Bought through live broadcast	Yes	400	100.00%
	No	0	0.00%
Gender	Male	162	40.50%
	Female	238	59.50%
Age	Under 18	30	7.50%
	18-30	153	38.25%
	30-45	171	42.75%
	Above 45	46	11.50%
Income	Under 500	2	0.50%
	500-1000	1	0.25%
	1000-1500	4	1.00%
	1500-2000	5	1.25%
	2000-2500	10	2.50%
	2500-4000	110	27.50%
	4000-6000	123	30.75%
	6000-8000	98	24.50%
	8000-10000	20	5.00%
	10000-12000	12	3.00%
	12000-14000	8	2.00%
	Above 14000	7	1.75%
Education	Under high school	55	13.75%
	High school	111	27.75%
	College	123	30.75%
	Bachelor	99	24.75%
	Above Master	12	3.00%
Frequency	1-2 times per month	21	5.25%
	3-5 times per month	270	67.50%
	6-9 times per month	89	22.25%
	10 or more times	20	5.00%

## 4. Data analysis

### 4.1. Reliability and validity analysis

This study utilizes SPSS 22 and Amos 22 for data analysis. The outcomes of the reliability and validity assessments are presented in Table 3. Table 4, on the other hand, displays the results of the distinguishing validity tests. In the context of this study, the variables P, H, A, and I stand for professionalism, homogeneity, attraction, and interactivity, respectively. Meanwhile, ET, PR, LE, and PI correspond to emotional trust, perceived risk, live-stream engagement, and purchase intention, respectively.

**Table 3:** Test results of reliability and validity (N = 400).

Dimensions	Item	Factor loading	Cronbach’s α	CR	AVE
Professionalism	A2	0.848	0.796	0.881	0.711
	A1	0.830			
	A3	0.851			
Homogeneity	B3	0.870	0.828	0.897	0.744
	B2	0.848			
	B1	0.870			
Attraction	C1	0.816	0.748	0.857	0.666
	C3	0.809			
	C2	0.824			
Interactivity	D3	0.848	0.793	0.879	0.707
	D1	0.824			
	D2	0.850			
Emotional trust	E2	0.881	0.842	0.905	0.760
	E3	0.854			
	E1	0.880			
Perceived risk	F2	0.846	0.853	0.927	0.717
	F3	0.872			
	F1	0.849			
	F5	0.824			
	F4	0.843			
Live-stream engagement	G2	0.820	0.864	0.902	0.699
	G4	0.826			
	G3	0.798			
	G1	0.896			
Purchase intention	H3	0.877	0.832	0.899	0.748
	H2	0.855			
	H1	0.863			

**Table 4:** Distinguishing validity test of model.

	P	H	A	I	ET	PR	LE	PI
P	**.842**							
H	.542**	**.863**						
A	.612**	.684**	**.816**					
I	.610**	.651**	.711**	**.841**				
ET	.630**	.703**	.742**	.751**	**0.872**			
PR	-0.026	-0.034	-0.077	-0.012	-0.063	**.846**		
LE	0.02	0.017	0.058	0.001	0.041	-.676**	**.835**	
PI	.572**	.656**	.681**	.706**	.759**	-0.088	0.065	**.866**

Note: The bold font on the diagonal is the square of AVE.

All items’ factor loadings are greater than 0.6. All CR values exceed 0.7. All AVE values exceed 0.5. They confirm the validity of the scale. Table 4 proves that there is no multicollinearity in this model. All the fit indices of the model were larger than the criteria value (see Table 5), which means a good fitness.

**Table 5:** Fit indices for CFA.

Index	X^2^	Df	X^2^/df	RMSEA	CFI	NFI	TLI
Criteria	/	/	<5	<0.08	>0.90	>0.90	>0.90
Value	254.728	80	3.184	0.074	0.946	0.924	0.929

To mitigate nonresponse bias and bolster the research’s validity, this study enhanced the survey’s attractiveness and ease of completion, thereby boosting the response rate [43]. A modest monetary incentive of 5 RMB was offered, and participants were assured of response confidentiality and anonymity to encourage honest and accurate answers.

To evaluate common method bias, a Harman single-factor test was performed. If a single factor explains over 50% of the covariance among variables, it suggests common method variance. However, the SPSS EFA results showed no single factor exceeding this threshold, indicating no dominant variance source.

To ensure the regression model’s reliability, multicollinearity was assessed using the Variance Inflation Factor (VIF). VIF values above 10 (or 5, depending on strictness) suggest multicollinearity issues. The linear regression analysis revealed all VIF values were below 5, indicating no significant multicollinearity in this study.

### 4.2. Direct and mediation effects

In this study, we methodically investigated the direct and mediating effect by utilizing Model 4 from the SPSS Process Macro, as developed by Hayes [45]. The direct effects, as detailed in Table 6, reveal significant insights. The professionalism of live streaming influencers exerts an influence on purchasing intentions, with a standardized path coefficient (β) of 0.165 and a p-value of 0.011, corroborating the research conducted by Liu, Wang [3], thereby lending credence to Hypothesis 1a. Furthermore, the homogeneity of live streaming influencers was found to affect purchasing intentions, with a β of 0.225 and a p-value less than 0.001, aligning with the conclusions drawn by Zhou and Tong [4], and thus validating Hypothesis 1b. The attraction of influencers also plays a role in influencing purchase intentions, as indicated by a β of 0.258 and a p-value less than 0.001, which is consistent with the outcomes reported by Zhong, Zhang [5], supporting Hypothesis 1c. Lastly, the interactivity of live streaming influencers significantly shapes consumers’ purchase intentions, evidenced by a β of 0.299 and a p-value less than 0.001. This finding is in agreement with the results presented by Sun, Gao [44], affirming the validity of Hypothesis 1d. The results, as depicted in Table 6, demonstrate that emotional trust acts as a mediator between the professionalism of live stream influencers and the willingness of consumers to make purchases. The beta coefficient (β) is notably significant at 0.440, with a p-value significantly below 0.001, thus reinforcing the findings reported by Zhong, Zhang [5] and validating Hypothesis 2a. Parallel to this, the mediating role of emotional trust is also observed in the relationship between influencer homophily and consumer purchase intention, with a β value of 0.383 and a p-value well below the threshold of 0.001. This finding is in harmony with the research conducted by Chen, Xie [29] and substantiates Hypothesis 2b. Moreover, emotional trust is identified as a mediator for the influence of live stream influencer attractiveness on purchase intention, with a β coefficient of 0.414 and a p-value less than 0.001. This corroborates the work of Liu, Wang [3] and supports Hypothesis 2c. Additionally, emotional trust is found to mediate the impact of influencer interactivity on consumer purchase intention, with a β value of 0.380 and a p-value below 0.001. This is in line with the research by Zhu, Li [7] and confirms Hypothesis 2d. The empirically-driven mediation analysis sheds light on the intricate link between the attributes of live stream influencers and the propensity of consumers to purchase, highlighting emotional trust as a pivotal intermediary.

**Table 6:** Direct effect and mediation effect.

Effect	Path	Effect-value	P-value	95% Confidence Interval	
				Lower	Upper
Total	P→PI	0.605	***	0.519	0.690
Total	H→PI	0.608	***	0.539	0.677
Total	A→PI	0.672	***	0.601	0.744
Total	I→PI	0.679	***	0.612	0.746
Indirect	P→ ET→ PI	0.440	***	0.360	0.525
Indirect	H→ ET→ PI	0.383	***	0.302	0.471
Indirect	A→ ET→ PI	0.414	***	0.325	0.509
Indirect	I→ ET→ PI	0.380	***	0.292	0.476
Direct	P→PI	0.165	0.002	0.078	0.250
Direct	H→PI	0.225	***	0.144	0.306
Direct	A→PI	0.258	***	0.167	0.349
Direct	I→PI	0.299	***	0.210	0.388

*Note:*

***p < 0.001.

### 4.3. Moderation effect test of education level and age

In order to explore the moderating effect of consumer education level and age, it is necessary to divide the subjects into higher education group and lower education group as well as older group and younger group. Higher education group (N = 111) includes ‘Bachelor’ and ‘Above Master’ while lower education group (N = 289) has ‘Under high school’, ‘High school’ and ‘College’. There are ‘30-45’ and ‘Above 45’ in the older group (N=217) while there are ‘Under 18’ and ‘18-30’ in the younger group (N=183).

As presented in Table 7, the data indicate that consumers with higher education exhibit a more pronounced influence on the P→PI and H→PI paths compared to those with lower education (0.205 versus 0.089; 0.242 versus 0.199, respectively). This finding suggests that consumer education level positively moderates the impact of live streaming influencer professionalism and homogeneity on purchase intention, indicating that the influence of these characteristics is more potent among more educated consumers. Conversely, the influence of consumer education level on the A→PI and I→PI paths is less pronounced for higher education compared to lower education (0.154 versus 0.230; 0.315 versus 0.329, respectively). This indicates a negative moderation, where lower levels of consumer education enhance the effect of live streaming influencer attraction and interactivity on purchase intention. In essence, consumers with lower education levels are more swayed by these influencer traits in their purchasing decisions. Consequently, Hypothesis 3a, which posits the moderating role of education level, is confirmed by these findings.

**Table 7:** Moderation effect of education level and age.

Path	Path coefficients			
	Higher education (N = 111)	Lower education (N = 289)	Older group (N=217)	Younger group (N=183)
P→PI	0.205** (0.103)	0.089* (0.052)	0.151** (0.066)	0.103 (0.064)
H→PI	0.242** (0.104)	0.199*** (0.048)	0.197** (0.065)	0.217*** (0.057)
A→PI	0.154 (0.109)	0.230*** (0.058)	0.177** (0.070)	0.277*** (0.073)
I→PI	0.315*** (0.085)	0.329*** (0.059)	0.310*** (0.068)	0.333*** (0.067)
ET→PI	0.775*** (0.056)	0.734*** (0.039)	0.768*** (0.040)	0.715*** (0.051)

Further analysis, as depicted in Table 7, reveals that the influence of age on the P→PI path is more pronounced among older consumers compared to their younger counterparts (0.151 versus 0.103). This indicates that age acts as a positive moderator in the relationship between the professionalism of live streaming influencers and consumer purchase intention, with older consumers being more significantly influenced by the professionalism of influencers in their buying decisions. Concurrently, it is observed that the impact of age on the H→PI, A→PI, and I→PI paths is less marked in older individuals than in younger ones (0.197 versus 0.217; 0.177 versus 0.277; 0.310 versus 0.333, respectively). This suggests a negative moderation effect, where younger consumers are more influenced by the homogeneity, attraction, and interactivity of live streaming influencers in their purchasing behavior. These findings substantiate Hypothesis 3b, which posits that consumer age plays a moderating role in the relationship between live streaming influencer characteristics and purchase intention.

The analysis presented in Table 7 indicates that the influence of the emotional trust to purchase intention (ET→PI) path is more pronounced among consumers with higher levels of education compared to those with lower education (0.775 versus 0.734). This observation implies that the educational background of consumers exerts a positive moderating effect on the relationship between emotional trust and purchase intention. Hence, Hypothesis 3c is supported. Similarly, the impact of the ET→PI path is found to be more substantial for older consumers than for their young counterparts (0.768 versus 0.715). This suggests that age also acts as a positive moderator in the influence of emotional trust on the willingness to purchase. Therefore, Hypothesis 3d is valid.

### 4.4. Moderation effect test of perceived risk and live-stream engagement

The SPSS Process Macro (Model 1), as developed by Hayes [45], was deployed to investigate the moderating impact of consumer perceived risk and live-stream engagement on the relationship between live streaming influencer characteristics and purchase intention. Data presented in Table 8 reveal that consumer perceived risk exerts a negative moderation on the links between live streaming influencer professionalism and attraction with purchase intention (β = -.0669, p =.0303; β = -.0596, p =.0230). This trend indicates that an increase in consumer perceived risk diminishes the influence of these influencer attributes on purchase intention.

**Table 8:** Moderation effect of perceived risk and live-stream engagement.

Hypothesis	Path	Unstd.	SE	P-value	Result
H3a	P×PR→PI	-.0669	.0308	.0303	Not Support
	H×PR→PI	-.0162	.0266	.5434	
	A×PR→PI	-.0596	.0261	.0230	
	I×PR→PI	-.0451	.0254	.0773	
H3b	P×LE→PI	.0870	.0406	.0329	Not Support
	H×LE→PI	.0209	.0359	.5614	
	A×LE→PI	.0726	.0356	.0420	
	I×LE→PI	.0665	.0343	.0530	

Conversely, consumer live-stream engagement operates as a positive moderator, enhancing the relationship between live streaming influencer characteristics—professionalism and attraction—and purchase intention (β =.0870, p =.0329; β =.0726, p =.0420). This outcome suggests that a higher level of engagement in live-streaming activities strengthens the propensity of consumers to be influenced by these characteristics in their purchasing decisions.

However, it was observed that neither consumer perceived risk nor live-stream engagement significantly moderated the path from live streaming influencer homogeneity and interactivity to purchase intention. The reason might be that the impact of the homogeneity and interactivity of live stream celebrities on consumer purchase intentions is fixed, and perceived risk and live-stream engagement do not affect these paths [4].

In summary, consumer perceived risk and live-stream engagement respectively play a negative and positive moderating role in the relationship between live streaming influencer professionalism and attraction and purchase intention. As such, Hypotheses 4a and 4b are invalidated.

## 5. Discussion

### 5.1. Theoretical contributions

Prior research has predominantly concentrated on examining the influence of a singular attribute of live streaming influencers on consumers’ propensity to purchase [1,3–5]. However, this investigation takes a holistic perspective, meticulously dissecting the collective impact of four pivotal attributes of live streaming influencers on consumers’ purchase intentions by integrating them into an integrated analytical construct, marking an innovative advancement in the research framework. It has been established in prior studies that emotional trust plays a crucial mediating role in shaping consumer purchase intentions in the realm of live streaming e - commerce [4]. Uniquely, this study pioneers the incorporation of emotional trust as a mediating factor within the context of how live streaming influencer traits impact consumer purchase intentions. This is mediation variable innovation. Moreover, disparities among consumers of varying ages and educational levels have been noted in influencer marketing [9]. This study further explores, for the first time, the influence of influencer characteristics on purchase intentions across different educational backgrounds and age groups in China in the context of live streaming e-commerce. Additionally, it provides novel insights into the nuanced ways in which emotional trust affects the purchase intentions of consumers with diverse educational attainments and age demographics. Lastly, while previous literature has acknowledged the role of perceived risk and engagement in influencer marketing [17], this study is the first to examine whether perceived risk and engagement moderate the relationship between influencer characteristics and purchase intentions in the context of live streaming e-commerce. These make significant contributions to influencer marketing theory.

### 5.2. Practical contributions

Manufacturers must recognize the pivotal role that live streaming influencers play in the realm of live streaming e-commerce. When selecting influencers for their campaigns, it is crucial to prioritize attributes such as their professionalism, homogeneity, attractiveness, and interactive capabilities.

During the live streaming process, influencers must be keenly aware of and actively cultivate the audience’s emotional trust which are crucial conduits through which influencers can effectively impact the audience’s purchase intentions. Moreover, influencers should be aware that the perception of emotional trust varies among consumers with different educational backgrounds and ages. For consumers with lower levels of education and younger audiences, influencers should more actively stimulate their emotional trusts, thereby increasing the willingness to purchase.

For viewers with higher levels of education, influencers should underscore the expertise and the homogeneity with consumers to resonate effectively. Conversely, when targeting less educated viewers, influencers should focus on enhancing their appeal and fostering more interactive experiences to captivate their audience. With older viewers, emphasizing professionalism can be particularly impactful, while younger viewers may respond more positively to influencers who cultivate a sense of belongings, attractiveness, and interactive engagement.

Beyond these considerations, to optimize the potential of influencer marketing, influencers should strive to minimize the perceived risk for their audience and maximize the level of engagement during live streams. Specifically, they need to foster a stronger emotional connection with viewers, extend viewing time of viewers, encourage following behavior of viewers, and increase the likelihood of monetary spending of viewers. Stakeholders should aim for excellence across all five dimensions—product quality, influencer performance, platform functionality, payment systems, and logistics—to create a seamless and satisfying experience for viewers. By focusing on enhancing these areas, influencers can decrease consumers’ perceived risk, drive greater viewer engagement and ultimately, realize more successful influencer marketing campaigns.

Policymakers should encourage live streaming platforms and agencies to strengthen professional training for live streamers, enhancing their knowledge and skill levels. At the same time, strict review mechanisms should be established to ensure the authenticity and reliability of live content, thereby increasing consumers’ trust in live streamers. Policymakers can also promote the development of more interactive features by live streaming platforms, such as real - time Q&A, voting, and lotteries, to enhance the interaction between live streamers and viewers. Additionally, live streamers should be encouraged to improve their personal charm and attractiveness to enhance viewers’ sense of identification.

For consumers with higher levels of education, policymakers should encourage live streaming platforms to provide more professional content and in - depth analysis to meet their rational decision - making needs. For consumers with lower levels of education, the focus should be on making the content more understandable and entertaining, reducing the complexity of information.

For younger consumers, policymakers can push live streaming platforms to add more entertainment and fashionable elements to attract their attention. For older consumers, the focus should be on the practicality and reliability of the content, providing more product usage instructions and after - sales service information. Policymakers should also promote the establishment of comprehensive risk management mechanisms by live streaming platforms, such as providing product guarantees, return policies, and user review systems, to reduce consumers’ perceived risks. At the same time, consumer education should be strengthened to improve their ability to identify and prevent risks.

Policymakers should encourage live streaming platforms and merchants to increase consumer participation in live streaming through various means, such as offering coupons, time - limited discounts, and interactive rewards. At the same time, live streaming technology should be optimized to ensure its smoothness and stability, enhancing consumers’ viewing experience.

## 6. Conclusions

Utilizing data collected from 400 questionnaires, this study investigated the mechanisms through which characteristics of live streaming influencers influence consumer purchase intentions, with a focus on consumer emotional trust as a mediating variable as well as consumer education level, age, perceived risk, and live-stream engagement as moderating factors. It is evident that all four live streaming influencer characteristics (professionalism, homogeneity, attraction, and interactivity) collectively influence consumers’ purchase intentions. Therefore, it is imperative that the industry recognizes the critical role that live streaming influencers play in the purchasing intention of consumers.

It is evident that the traits of live streaming influencers have a positive effect on consumers’ intent to purchase and the emotional trust mediates the influence of live streaming influencer characteristics on purchase intention. It is clear that consumers’ educational level positively moderates the relationship between the professionalism and homogeneity of influencers and their purchase intentions, while it negatively moderates the relationship between influencers’ attraction and interactivity with purchase intentions. Additionally, the age of consumers positively moderates the link between the professionalism of influencers and purchase intentions, but negatively moderates the links between homogeneity, attraction, and interactivity of influencers and purchase intentions. Furthermore, both consumers’ educational level and age positively moderate the impact of emotional trust on purchase intentions. Lastly, perceived risk and live-stream engagement respectively exert negative and positive moderating effects on the influence of influencer professionalism and attraction on purchase intentions.

Therefore, it is important to prioritize attributes such as professionalism, homogeneity, attractiveness, and interactive capabilities when selecting influencers for live streaming. Furthermore, influencers should strive to minimize the perceived risk for the consumers and maximize the level of engagement during live streaming.

This study, while offering valuable insights, acknowledges some limitations that merit attention. Firstly, in examining moderating effects, the focus was exclusively on the impact of consumer education level, age, perceived risk, and live-stream engagement. However, other factors, such as the characteristics of products endorsed by live streaming influencers and the specific promotional tactics they utilize, could also significantly shape consumers’ purchase intentions. These aspects are ripe for further exploration in future research. Secondly, the influence of live streaming influencer characteristics over time, including potential variations across different seasons or years, remains uncharted. The dynamic nature of consumer behavior and market trends suggests that the impact of influencer traits may evolve over time, which could be explored in subsequent research.

## Supporting information

S1 File Data. (XLSX)
